# Multiscale harmonization and semantic integration of biomedical data enable biological insights through immersive exploration

**DOI:** 10.64898/2026.07.07.737090

**Published:** 2026-07-11

**Authors:** Andreas Bueckle, Chenchen Zhu, Alex Y. H. Wong, Archibald Enninful, Yang Miao, Negin Farzad, Maria Pedersen, Courteney Mattison, Nicholas Sloan, Jason Mares, Cheng Xing, Bruce W. Herr, Juhi Khare, Yash Kumar, Keyur Parekh, Siddhi Chavan, Paean Luby, Ushma Patel, John Hickey, Gary D. Bader, Hemali Phatnani, Vilas Menon, Rong Fan, Peter K. Sorger, Michael Snyder, Katy Börner

**Affiliations:** 1 Department of Intelligent Systems Engineering, Indiana University, Bloomington, IN, USA; 2 Department of Genetics, Stanford University, Stanford, CA, USA; 3 Laboratory of Systems Pharmacology, Harvard Medical School, Boston, MA, USA; 4 Ludwig Centre at Harvard, Harvard Medical School, Boston, MA, USA; 5 School of Engineering & Applied Science, Yale University, New Haven, CT, USA; 6 Department of Biomedical Engineering, Duke University, Durham, NC, USA; 7 Center for Genomics of Neurodegenerative Disease, New York Genome Center, New York, NY; 8 Department of Neurology, Columbia University Irving Medical Center, New York, NY; 9 The Donnelly Centre, University of Toronto, Toronto, Ontario, Canada; 10 Department of Molecular Genetics, University of Toronto, Toronto, Ontario, Canada; 11 Princess Margaret Research Institute, University Health Network, Toronto, Ontario, Canada; 12 Department of Computer Science, University of Toronto, Toronto, Ontario, Canada; 13 Lunenfeld-Tanenbaum Research Institute, Toronto, Ontario, Canada; 14 Canadian Institute for Advanced Research (CIFAR), Toronto, ON, Canada; 15 Center for Translational and Computational Neuroimmunology, Department of Neurology, Columbia University Irving Medical Center, New York, NY; 16 Berlin Institute of Health at Charité, Universitätsmedizin Berlin, Berlin, Germany

## Abstract

The Human Reference Atlas (HRA) enables multiscale data exploration and visualization. We present “**HRA: Powers of Ten,**” a virtual reality (VR) application for integrating, harmonizing, and visualizing data within the **HRA Organ Gallery**. It enables immersive navigation from a whole-body view of 81 organs to datasets across 5 organs, 5 assay types, and 4 spatial scales using a Multiscale Elevator System. The application, data, and code are available open-source.

Single-cell atlassing portals such as the Human BioMolecular Atlas Program (HuBMAP)^1,2^ and the Cellular Senescence Network (SenNet)^3^ publish datasets across scales for multiple organs of the healthy adult human body for both sexes, multiple ethnicities, and across adult ages. The Canadian Institute for Advanced Research (CIFAR) MacMillan Multiscale Human (cifar.ca/research-programs/cifar-macmillan-multiscale-human) effort aims to build an explorable model of the human body across scales to drive advances in medicine and therapeutics. The data from these efforts can be interrogated semantically and spatially but only on two-dimensional (2D) screens with limited three-dimensional (3D) affordances^4^. Imagine, for example, examining the 3D location of a cell in the context of a tissue, inside of an organ, and as part of a human body— across multiple orders of magnitude. To be tackled effectively, 3D multiscale data and inquiries require 3D user interfaces (UIs).

In support of multiscale harmonization, the HRA^5^ provides terminologies and data structures for specimens, biological structures, and spatial positions of experimental datasets inside 3D reference organs of the adult human body^6^. Building on it, prior work has showcased the HRA Organ Gallery^7^, a VR application that lets users explore the HRA in immersive 3D. Here, we introduce “**HRA: Powers of Ten**,” a data integration and visualization application that uses the HRA Common Coordinate Framework (CCF)^6^ to harmonize, visualize, and explore data across 5 organs (lymph node, brain, large/small intestine, liver), 5 assay types (CO-Detection of IndEXing [CODEX]^8^/PhenoCycler, Visium [www.10xgenomics.com/platforms/visium], v-CyCIF^9^, Xenium^10^, and serial block-face scanning electron microscopy [SBF-SEM]^11^), and and 4 scales (meters to centimeters to millimeters to microns). It features a **Multiscale Elevator System** (see Extended Data Fig. 1) where users can traverse the human body like an inverted skyscraper, with the Whole Body level at the top and each subsequent elevator floor shrinking the user by 1 order of magnitude, see details under “HRA: Powers of Ten’s Multiscale Elevator System” below. A total of 8 scenes are presented in the application; each contains data from a different Data Expert, all co-authors on this paper. The scenes are named after the effort from where the data originates (e.g., “hubmap”), the organ (“large_intestine”), the name(s) of the Data Expert(s) (“wong”), the prevalent metric scale (“100_microns”), and the same metric scale expressed as a power of 10, i.e., its Multiscale Elevator System floor (“10_4”). Data Experts explain biological insights under “Insights enabled by multiscale integration and exploration in VR.” The process of adding data to the HRA Organ Gallery is described under “Validate method performance and demonstrate its general applicability” and in a standard operating procedure (SOP)^12^.To enable readers without VR headsets to understand what data is presented in each scene, videos featuring voice-overs by the Data Experts and other relevant metadata are available at cns-iu.github.io/hra-organ-gallery-supporting-information/#videos. Fig. 1 provides a high-level gallery view of all 8 scenes. Extended Data Fig. 2 provides detailed screenshots with illustrations of users. Implementation details with additional **Extended Data Figures** are provided in the Methods section. As part of the HRA Organ Gallery in VR, HRA: Powers of Ten is available for free through the Meta Store at www.meta.com/en-gb/experiences/hra-organ-gallery/5696814507101529 to over 20 million users owning affordable, portable, off-the-shelf Meta Quest headsets.

The development of 5 of the 8 scenes in this application was conducted collaboratively by 7 early-stage investigators (ESIs) and partially funded by a co-author Bueckle’s HuBMAP JumpStart Fellowship (hubmapconsortium.org/jumpstart-program/#andreas2024), awarded by the National Institutes of Health (NIH). All 7 ESIs are co-authors on this paper (Bueckle, Zhu, Wong, Enninful, Miao, Farzad, Xing); 4 served as Data Experts (scene names have ESI names in them); co-author Bueckle implemented all with his team as the Organ Gallery Lead (development lead). This work was conceived of during a JumpStart-funded workshop, see **Supplementary Note 1** and cns-iu.github.io/workshops/2024-10-24-jumpstart-workshop. 5 of the ESI authors attended this workshop.

## HRA: Powers of Ten’s Multiscale Elevator System:

**Extended Data Fig. 1.**
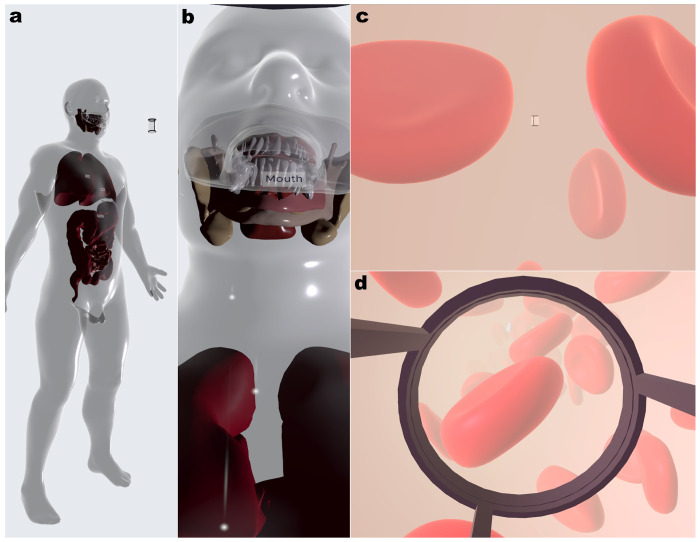
The 2 reference objects for the Elevator Transition Scenes while traveling to the 10^−2^ (**a-b**) and 10^−7^ levels (**c-d**).

To explore the human body, 10 orders of magnitude must be traversed in 3D, from the whole body down to proteins. HRA: Powers of Ten makes these accessible via a **Multiscale Elevator System** where the body is envisioned as an inverted skyscraper with an elevator taking the user from the Whole Body on the top floor down. Each level constitutes an order of magnitude scale change. Each dataset is visualized in its own *scene*, i.e., a closed 3D immersive space that the user can navigate, which is situated on a *level*, i.e., a discrete zoom magnification. Each level gets its “floor number,“ or **power**, i.e., −4 in 10^−4^, from the exponent to base 10 of the scale of the dataset(s) featured. For example, in level 0, the Whole Body level, 1 meter in physical space corresponds to 10^0^ (=1) meters in virtual space, i.e., no magnification is applied. Likewise, in level −4, the Cell Groups level, 1 meter in physical space corresponds to 10^−4^ meters (=100 microns) in virtual space, which means a magnification of 4 orders of magnitude. Because this is a VR application, the user always has their own body as a reference, e.g., their extended arms. A level can have multiple scenes; for example, the 10^−4^ level has 5 scenes with data from 4 organs (lymph node, small intestine, large intestine, liver). To access them, a virtual Elevator Panel is featured in every scene that allows the user to move on to a new scene (see Methods section and Extended Data Fig. 3.d). To further communicate scale differences between scenes, each level features an Icon of a real-world object, which is visible in the Skybox, i.e., the cube map texture that gives the illusion of a spherical sky, see Methods section and Extended Data Fig. 4. When switching to a scene on a different level, an optional Elevator Transition Scene is played where the user is placed inside a virtual elevator box and either sees the entire human body or, in lower scales, a collection of red blood cells as reference models given the appropriate scale difference (see Extended Data Fig. 1 and the last video listed under cns-iu.github.io/hra-organ-gallery-supporting-information/#videos).

## Insights enabled by multiscale integration and exploration in VR:

The 8 scenes presented in this paper are spread across 4 levels: the Whole Body level with 81 3D reference organs of the HRA v2.5 (10^0^, 1 meter), Small Organs (10^−2^, 1 centimeter), Large FTUs (10^−3^, 1 millimeter), and Cell Groups (10^−4^, 100 microns). Each scene gets its name from (a) the funded effort that the data originates from, (b) the organ, (c) the last name of the Data Expert(s), and (d) the level at which the data was integrated.

**Fig. 2.**
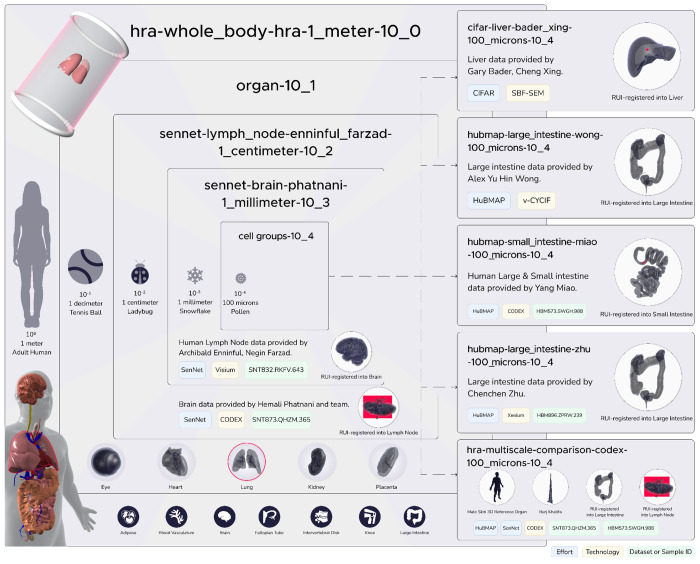
Metadata for the 8 scenes in this paper in the context of the Multiscale Elevator System of HRA: Powers of Ten.

Fig. 2 presents details on the datasets used in HRA: Powers of Ten scenes. Implementation details for all scenes are provided in the Methods section.

## hra-whole_body-hra-1_meter-10_0 *(a whole body introduction to the HRA)*:

**Extended Data Fig. 2.**
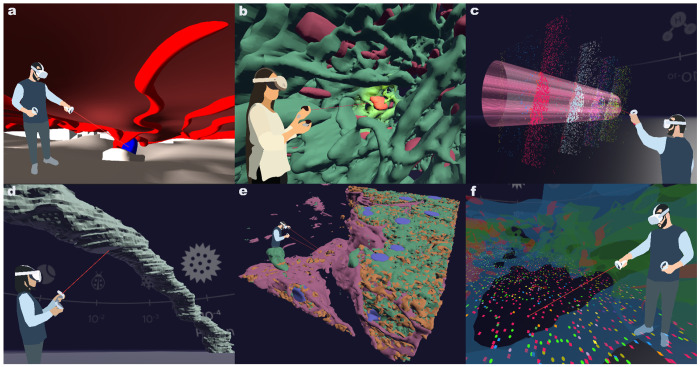
A tour of HRA: Powers of Ten. **a**, The maternal interface of the placenta with the umbilical cord being formed by blood vasculature in hra-whole_body-hra-1_meter-10_0 after the user has scaled up the organ. **b**, The user inspects the CD8 cells with ID 72 in the cropped volume (light-sheet/v-CyCIF) in hubmap-large_intestine-wong-100_microns-10_4. **c**, The user places a search cylinder over the 4 aggregation layers in hubmap-small_intestine-miao-100_microns-10_4. **d**, The user points at the bile duct, enlarged 20x, in cifar-liver-bader_xing-100_microns-10_4. e, In the same scene, the user explores a 3D SBF-SEM volume with cell nuclei (blue), plasma membrane (pink), endoplasmic reticulum (green), and mitochondria (orange). **f**, The user inspects cell nuclei and RNA molecules on top of a 3D reconstruction of Xenium data from a polyp in the large intestine in hubmap-large_intestine-zhu-100_microns-10_4.

As of July 2026, the start scene of the HRA Organ Gallery contains 81 3D reference organs of HRA v2.5 alongside 2,762 tissue blocks representing 13,344 datasets from 1,021 donors, registered by 54 tissue data providers with the Registration User Interface (RUI)^4^. Using a virtual keyboard with Icons, the user can select an organ (plus sex and laterality if available) to load a high-resolution version of a 3D organ to move, rotate, and scale while inspecting the tissue blocks in their native (or relative) size. They can also inspect anatomical details in the organ (such as the umbilical cord shown in Extended Data Fig. 2.a). A detailed view is available in Extended Data Fig. 5.

## sennet-lymph_node-enninful_farzad-1_centimeter-10_2 *(spatial patterns of senescent cells)*:

Interactions between senescent^3^ cells in human lymph nodes are easy to miss in 2D. HRA: Powers of Ten features a CODEX dataset^13^ obtained from a 86-year old male donor with SenNet ID SNT832.RKFV.643 featuring 36,964 cells of 16 cell types shown (see **Supplementary Fig. 1**). The scene illustrates that lymph nodes are highly structured tissues with distinct cellular regions, including B-cell-rich follicles and T-cell-dominant interfollicular zones. Additional immune cells, such as macrophages, monocytes, and dendritic cells are distributed in spatial patterns that reflect their roles in immune surveillance and response. This enables a more intuitive 3D exploration by making their spatial patterns and interactions with other cells within their microenvironment explicit. Further, this immersive approach makes it easier to examine the relationship between immune cells and vasculature as well as stromal cells in the lymph node. A brush and brush-and-link function to highlight all occurrences of a cell type across the tissue, the user can map individual immune cells and their spatial relationships within the tissue. Distinct anatomical regions are visible, including B cell-rich follicles and T cell-dominated interfollicular zones, along with macrophages, dendritic cells, and vascular cells. Looking ahead, VR holds promise for deeper understanding of complex tissue microenvironments particularly within the context of cancers. With spatial proteomic data generated from serial tissue sections, there is hope to construct full 3D atlases of lymph nodes to study lymphomas (specifically Diffuse Large B Cell Lymphoma^14^ and Angioimmunoblastic T Cell Lymphoma^15^) in unprecedented detail via HTAN (see humantumoratlas.org/center/hta209). VR can help visualize tumor cells and track their evolution within the tumor microenvironment. A detailed view is available in **Supplementary Fig. 1**.

## sennet-brain-phatnani-1_millimeter-10_3 *(simultaneous comparison of 8 senescence hallmarks in 3D space)*:

The scene features a Visium slide of dorsolateral prefrontal cortex tissue from the brain of a 74-year-old female donor (dataset ID SNT873.QHZM.365). It contains a 3D spike plot with color-coded senescence hallmark expressions for senescent cells. The capture area of the Visium slide is 6.5 x 6.5 millimeters, magnified by 3 orders of magnitude to 6.5 x 6.5 meters. There are a total of 4,992 spots per capture area; each spot is 55 microns in diameter with a 100 microns center-to-center distance between spots. Each spot represents gene activity from small cell groups, visualized with colored lines indicating levels of aging-related gene signatures. Different colors correspond to curated gene sets representing 8 senescence hallmarks: Resistance.to.Apoptosis, DDR, SASP, Cell.Cycle.Arrest (see related publication^16^ for details on these four); Fridman^17^; Activated.p.Targets^18^, Senmayo^19^; and those curated by the San DiegoTissue Mapping Center. Lines above or below the plane of a cell spot show enrichment or depletion of these genes. This visualization helps reveal spatial patterns and relationships in senescence.

Identifying patterns, trends, and outliers in these 39,936 spatially explicit expression markers is non-trivial. Using data visualizations such as the 3D spike plot presented here, users can investigate spatial patterns that may indicate subclinical senescence, emerging senotypes, or localized senescence microenvironments (SMEs). Do zones of elevated SASP border regions with early DDR or cell-cycle arrest? Do these arrangements indicate tissue-specific aging pathways? Through immersive, multi-hallmark research, this scene enables hypothesis generation and the development of computational models that capture spatial relationships not easily detectable in 2D. Additional 2D plots enable auxiliary data visualization with scatter graphs with individual senescence hallmark values. A detailed view is available in Extended Data Fig. 6.

## hubmap-large_intestine-wong-100_microns-10_4 *(Volumetric cyclic immunofluorescence (v-CyCIF)*^9^ *reveals 3D topology of cells and surrounding nerves in clinical specimens)*:

How can multiscale data with many different formats (tabular, nested/hierarchical, 2D, 3D) be integrated into a coherent exploration and analysis space^20^? How can this inherently static data “come alive,” become more interactive, and multi-modal for the user? To explore these questions, this scene features 2 complementary volumetric datasets from the human large intestine. The first is a light-sheet^21^ microscopy image crop featuring ~200 CD8^+^ conventional T cells (Tcon) and βIII-tubulin expressing (TUBB3) enteric nerves. The second is a high resolution confocal dataset with blood vessels (CD31, endothelial cells), epithelial cells (Claudin-7), in addition to T cells and nerves. In the light-sheet dataset, CD8^+^ Tcon cells and nerve structures are mapped in 3D, which shows most immune cells in direct physical contact with nerves, while some migrate towards the lumen. Users can interactively inspect cells by grabbing, moving, and rotating them in 3D using their VR controllers; they can visualize properties (distance to nearest nerve ending, volume, minimum intensity, and sphericity) via a heatmap. As a result, HRA: Powers of Ten uses VR visualization to transform static image volumes into immersive, manipulable representations of tissue organization. Together, this scene combines the imaging depth of light-sheet microscopy with the spatial resolution of confocal microscopy, which enables multiscale analysis of intact tissue architecture. The approach uses v-CyCIF^9^ to integrate multiple markers, which enables detailed spatial analysis of immune, epithelial, and vascular structures. In 2D, the 3D morphology of individual cells remains obscured by their proximity within dense cellular neighborhoods. VR supports an “exploded view” that greatly enhances inspection capabilities. Users can virtually separate and examine 3D T cells in detail and discard cells with poor segmentation quality. A detailed view is available in Extended Data Fig. 7 and Extended Data Fig. 2.b.

## hubmap-small_intestine-miao-100_microns-10_4 *(layered presentations of cellular neighborhoods)*:

The complexity of multi-scale cellular neighborhoods and tissue organization^22,23^ in the human body is difficult to appreciate, understand, and interpret in static views. HRA: Powers of Ten enables researchers to visualize and overlay cellular neighborhoods across multiple levels of aggregation, i.e., from the local microenvironment to entire tissue units. This scene features a VR visualization of a representative small-intestine tissue region^22^ acquired using CODEX (dataset ID HBM573.SWGH.988). The tissue is displayed across 4 ascending levels of aggregation: cell type, neighborhood, community, and tissue unit, thus, the same cell is represented across 4 interconnected levels of aggregation. At the cell-type level, individual cells are shown by their annotated identities. At higher levels, cells are grouped based on the composition of their surrounding neighbors, with each scale using a different neighborhood range to reveal tissue organization at increasing spatial breadth. At the neighborhood scale, compartmentalized local microenvironments that support specific tissue functions are captured. The broadest scale, tissue unit, aligns with known intestinal histology, spanning structures from the mucosa to the muscularis externa. The visualization of different scales allows users to explore finer details, e.g., identifying CD4^+^ and CD8^+^ T cells within unique multicellular environments. An alluvial diagram further enables simultaneous multi-scale analysis and quantitative network visualization by linking cellular distributions across levels. Additionally, an interactive selection cone that highlights the same cell across different aggregation levels further enhances spatial interpretation and enables users to connect local cellular identity with higher-order tissue architecture. This scene features 3,252 cells at 4 aggregation levels. A detailed view is available in Extended Data Fig. 8 and Extended Data Fig. 2.c.

## hubmap-large_intestine-zhu-100_microns-10_4 *(integrating 3D tissue, 2D cells, and RNA molecules into a coherent, multiscale zoom)*:

Due to their flatness, 2D visualizations can only capture so much detail about the stereotyped organization of tissue. By creating an immersive environment that enables visualization across multiple biological scales (tissues, cells, and molecules), VR helps users make more informed identifications of cell types and better understand putative crosstalks between different cell types, e.g., the co-localization of signaling molecules and ligand-receptor pairs, particularly among diverse tissue-resident immune cells. This enables scientists to better assess and refine their 3D tissue reconstruction models. This HRA: Powers of Ten scene features a 3D model of a human pre-cancerous large intestine polyp (sample ID HBM896.ZPRW.239). The scene integrates 3 data types: (1) a 3D reconstruction of the polyp from 29 serial thin sections were assayed using 10x Xenium^10^ assay, a highly multiplexed spatial method for identifying RNA molecules *in situ*; the reconstruction was achieved with Space-map^24^, a method optimized for reconstructing 3D maps from serial tissue sections, to generate the anatomically faithful 3D model presented here; (2) the same 29 layers as lists of 2D cells with x-position, y-position, and cell type labels; and (3) RNA molecules as a CSV file with 3D coordinates and identifiers. The cells were stained with Xenium to identify cell location as well as gene expression. Key cell types include cancer-associated fibroblasts, CD4^+^ T cells, and enterocytes. Users can zoom from the overall tissue structure down to individual cells and molecular signals, where the presence of RNA molecules indicates cell identity and activity. Regions with dense molecular signals may suggest proliferative, potentially pre-cancerous cells.These datasets are synthesized into an integrated model that represents an approximately thousand-fold difference in scale, comparable to the difference between a basketball and a grain of sand. This integrated 3D approach enables analysis of cell interactions and spatial patterns for offering insights into early cancer development and the organization of the tissue microenvironment. This scene features 18,284 cells on layer 16 with 20,918 surrounding RNA molecules in 3D. A detailed view is available in Extended Data Fig. 9 and Extended Data Fig. 2.f.

## cifar-liver-bader_xing-100_microns-10_4 *(allow 3D exploration and validation of liver data)*:

This scene features human liver tissue and highlights the 3D architecture of the periportal region using SBF-SEM at nanometer resolution^25^. Shown are 3 anatomical landmarks: the portal vein, the bile duct, and hepatocyte organelle structures (on a subcellular scale) with mitochondria, cell nuclei, plasma membranes, and the endoplasmic reticulum. Approximately fifty hepatocytes are segmented, each enclosed by plasma membranes that define distinct cellular compartments. The user can grab, move, rotate, and scale each 3D model. The models were preprocessed using Blender (www.blender.org ), an open-source 3D modeling tool, and Microscopy Nodes^26^ (extensions.blender.org/add-ons/microscopynodes). A detailed view is available in Extended Data Fig. 10 and Extended Data Fig. 2.d–e.

## hra-multiscale-comparison-codex-100_microns-10_4 *(allow scale-accurate comparison of datasets from lymph node and small intestine in 3D space)*:

Most scenes in the HRA Organ Gallery focus on 1 dataset (or a collection thereof) from 1 organ. When entering this scene, however, the user sees 4 entities with their true scale differences: the small intestine dataset HBM573.SWGH.988 from hubmap-small_intestine-miao-100_microns-10_4, the lymph node dataset SNT832.RKFV.643 from sennet-lymph_node-enninful_farzad-1_centimeter-10_2, the HRA 3D reference organ for the male right kidney, and the HRA 3D reference organ for the male skin from hra-whole_body-hra-1_meter-10_0. Additionally shown is a 3D model of the tallest building on Earth (Burj Khalifa, 882 meters).

These entities coexist in a shared space, see **Supplementary Fig. 2**. Typically, in each scene, datasets are scaled to about 1 meter in physical space. In this scene, however, the 2 datasets and the 3D reference organ are shown with the correct scale difference, where the small intestine data is 1 meter wide (see **Supplementary Fig. 2.d**), the lymph node data is 100 meters wide (two powers of 10 larger, see **Supplementary Fig. 2.c**), and the human body is 10,000 meters tall (four powers of 10 larger, see **Supplementary Fig. 2a-b**). The 3D model of Burj Khalifa, however, is static and displayed in its real physical scale (828 meters); to illustrate this, it is rendered in red. As a result, when the scene starts, the skin 3D reference organ is already barely visible. For the small intestine and lymph node data, cell type groupings include immune cells, mesenchymal cells, and endothelial cells, which highlights shared biological components across tissues despite scale differences. Re-using existing work^27^, data preprocessing aggregated similar cell types into higher level categories, enabling clearer cross tissue comparisons. The user can dynamically resize the small intestine dataset and the lymph node and skin will scale concurrently, while Burj Khalifa provides a static, consistent large-scale benchmark (see see **Supplementary Fig. 2.b**). This multiscale visualization demonstrates how biological structures of vastly different sizes can be compared within a unified spatial framework. A detailed view is available in **Supplementary Fig. 2**.

## Validate method performance and demonstrate its general applicability:

A SOP^12^ details how new datasets can be harmonized into the HRA Organ Gallery. It outlines 2 data categories: 3D models (e.g., OBJ, FBX, or glTF from v-CyCIF^9^ or and SBF-SEM^11^, OME-TIFF via STL, OME-ZARR) and data points (CSV, XSLX from CODEX^8^, Xenium^10^, Visium [www.10xgenomics.com/platforms/visium], or SWC^28^) and assigns responsibilities to predetermined roles in the process: the Data Expert (who provides the data), the Designer (who ensures aesthetic fit), the Developer (who implements new scenes), and the Organ Gallery Lead (who coordinates all).

## Methods

### Background

#### HRA:

**Powers of Ten** addresses an important gap in biomedical visualization. While commercial applications for viewing the human body are widely used to teach anatomy, none support multiscale data exploration from the whole body to (sub-)cellular resolution. All of these scales are critical to understanding human physiology. 3D stereoscopy, supported by increasingly high-resolution displays in VR headsets, enables accurate perception of depth and distances between different organs, cells, and subcellular entities (~2k pixels per eye for the Meta Quest 3 used in the HRA Organ Gallery); these also allow detailed views of spatial arrangements and size comparisons.

Further, natural input via VR controllers supports advanced interactions with data beyond traditional mouse and keyboard paradigms, e.g., grabbing, moving, rotating, and scaling cells and tissue regions with the user’s own hands. These effects are further enhanced by 2 features of the HRA Organ Gallery: 3D scale boxes (instead of 2D scale bars) with easy-to-recognize scale-specific objects as landmarks and virtual Elevator Transition Scenes to communicate scale changes (see Shared features between scenes section for details). Where multiscale visualization has been implemented, it’s been so within a limited scope. The vast majority of these applications are focused on genomics and allow users to compare genomes of varying size and phylogenies^29,30^. Some genome-oriented multiscale applications are more visualization-focused, such as the Multiscale Unfolding application that takes users from a genome’s chromosomes to its double helices and base pairs^31^. Multiscale visualizations are appreciably less common at the organ-level, and where they do exist they are frequently limited in their scope. For example, one VR prototype allows users to view a 3D mouse prostrate at the organ and sub-organ levels, with an additional quantitative histology level^32^. Another leveraged computed tomography to allow users to move from the whole organ scale down to the ion channel level with select muscles and bones^33^.

Prior work has also explored a variety of approaches for communicating and navigating scale, both within biological datasets and in broader educational and visualization contexts. For example, some have advocated for a Google Maps–like navigation paradigm where higher-resolution datasets are embedded within larger anatomical contexts and allow users to progressively zoom into regions of interest while maintaining spatial awareness across scales^33,34^. 2D experiences like the Nikon Universcale (www.nikon.com/company/corporate/sp/universcale/?ref=1), Kees Boeke’s influential illustrations in “Cosmic View: The Universe in Forty Jumps”^35^, and the ”Powers of Ten” educational movie^36^ let users view objects across scales, but these are limited to non-stereoscopic web applications, static pages in a book, and non-interactive video.

### Validation via SOP

The SOP^12^ entitled “Adding New Datasets to the Human Reference Atlas Organ Gallery” outlines the process for submitting 2D and 3D datasets for harmonization and visualization with the HRA Organ Gallery. It uses Jupyter Notebooks, Python, and Blender. The primary audience is Data Experts. The HRA Organ Gallery was developed with Unity 2022.3 (unity.com), a 3D real-time development platform. The Shared scripts and Unity features between scenes section contains more information about native and custom Unity functionality used during development.

#### Roles and responsibilities:

4 key roles govern the workflow. The **Data Expert** provides raw datasets and domain knowledge. The **Organ Gallery Lead** oversees the development of the HRA Organ Gallery and serves as the primary point of contact. The **Designer** creates VR scene specifications based on discussions between the Data Expert and the Organ Gallery Lead. Finally, the **Unity Developer** processes the submitted data and builds finalized scenes based on the Designer’s specifications under guidance from the Organ Gallery Lead. The Data Expert approves the final build of the scene.

#### Engagement:

Typically, the Organ Gallery Lead initiates the collaboration by contacting the Data Expert via email. Upon engagement, the Organ Gallery Lead assigns the Data Expert a limited-access Google Drive folder for secure file uploads and a dedicated Acknowledgements document for capturing metadata, special handling requirements, and attribution information. Data Experts are expected to complete all required fields in this document before their scene is finalized and released.

#### Data formatting requirements:

The SOP specifies formatting requirements depending on data type. For cell location and annotation data derived from CODEX^8^, Xenium^10^, and v-CyCIF^9^, datasets must be structured with columns for x, y, and optionally z coordinates, alongside a cell type or biological entity column. Preferred submission formats are 3D models (e.g., OBJ, FBX, or glTF from v-CyCIF or and SBF-SEM^11^, OME-TIFF via STL, OME-ZARR) and data points (CSV, XSLX from CODEX, Xenium, Visium [www.10xgenomics.com/platforms/visium], quantification tables for v-CyCIF, or SWC^28^). Blender is used for pre-processing if needed; otherwise, these files can be used in Unity right away. Custom processing for OME-ZARR is also completed and documented in the SOP^12^ but not featured in the version of the HRA Organ Gallery documented in this paper.

### Shared features between scenes

**Extended Data Fig. 3.**
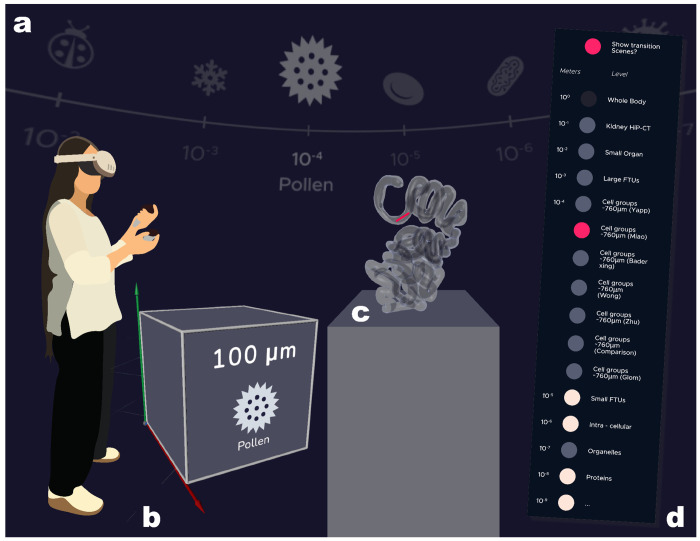
Shared features between scenes. **a,** Skyboxes with Icons of real-world objects. **b,** A Scale Cube with the Icon and metric scale of the scene (pollen, 10^−4^, 100 microns), and the name of the Icon spelled out. **c,** An Extraction Site Component for a tissue block (sample: HBM787.FHZM.948, dataset: HBM573.SWGH.988) in the small intestine from hubmap-small_intestine-miao-100_microns-10_4. **d,** An Elevator Panel allowing users to travel to scenes across the human body.

Each scene has a core set of features to enable multiscale navigation and exploration: an *Elevator Panel*, a *Scale Cube*, a *Skybox and Icon*, and an *Extraction Site Component*. Extended Data Fig. 3 shows all 4 shared features together.

#### Elevator Panels

Every scene contains an Elevator Panel (see Extended Data Fig. 3.d), which allows the user to access all other scenes via buttons; each scene has a level number on which it is placed (expressed as a power of ten) and a name. If there are multiple scenes on the same level (such as on 10^−4^ = 100 microns), the level number is only displayed once. If a level has no scenes in it yet, it is colored white (locked), e.g., as of July 2026, the 10^−6^ level. If a scene can be entered, its button is colored HRA Blue (#201E3D). If it is active, i.e., if the user is currently in it, it is colored HRA Red (#FF0043). Both colors come from the HRA Style Guide^37^ and HRA Design System (docs.humanatlas.io/dev/design-system). At the very top of the Elevator Panel, a toggle button lets the user turn Elevator Transition Scenes on and off (see Elevator Transition Scenes section below). The Elevator Panel is instantiated in all scenes.

#### Scale Cubes

While the scale of the virtual environment changes depending on the level the user travels to, the scale of the physical environment that the user is physically located in is, of course, persistent. As a result, the mapping between virtual and physical environments changes. To maintain a reference to the physical environment, a Scale Cube (see Extended Data Fig. 3.b) with a volume of 1 cubic meter enables the user to get a visual reference for the length of 1 meter in the physical environment while in the virtual environment. On its user-facing side, the Scale Cube displays the current size that 1 meter in the physical environment corresponds to in this scene (e.g., 100 μm in Extended Data Fig. 3.b) as well as the Icon for that level (e.g., a pollen, see Skyboxes and Icons section below) and the name of the object that the Icons depicts (e.g., “Pollen”). The object on the Icon represents everyday objects that are understandable without domain expertise. The Scale Cube auto-rotates around its y-axis to always face the user.

#### Skyboxes and Icons

**Extended Data Fig. 4.**
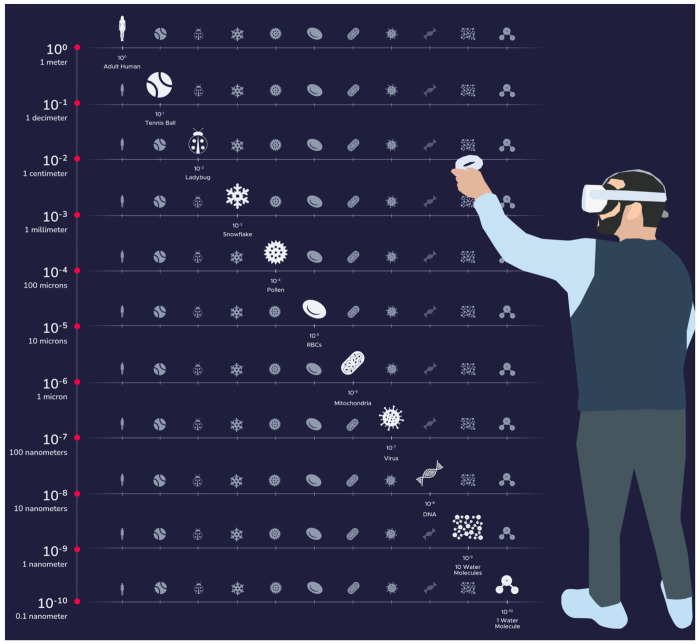
Skyboxes for scenes on every level show a numbered line with the power (10^x^) and a representative Icon for an object at that scale. When laid out next to each other, they look like constellations in the night sky.

Skyboxes (see Extended Data Fig. 3.a and Extended Data Fig. 4) are large-scale textures (typically cubemaps) commonly used in 3D environments that simulate a sky environment by wrapping around an entire scene (docs.unity3d.com/2022.3/Documentation/Manual/skyboxes-using.html). In the HRA Organ Gallery, Skyboxes communicate scale transitions and to support user orientation during navigation across biological levels. Their primary function is to serve as large-scale environmental cues to reduce visual emptiness and help users contextualize their relative size in each level. The conceptual foundation for the Skyboxes is the Nikon Universcale (http://www.nikon.com/company/corporate/sp/universcale) and the “Powers of Ten” movie^36^.

All Skybox illustrations were designed in Figma (www.figma.com). The design approach emphasized clean vector shapes to maintain legibility at large viewing distances. Colors were derived from the HRA Style Guide^37^ and HRA Design System (docs.humanatlas.io/dev/design-system) for stylistic continuity across light and dark modes. Subtle luminance gradients were introduced to create a sense of atmospheric depth and to avoid the flat appearance typical of uniform backgrounds. Each Skybox was produced at high resolution to reduce stretching and pixelation when mapped to a spherical projection. The vertical placement of objects above the horizon was also determined through trial and error. Object height was tested both in the spherical Skybox in VR and in the flat 2D rectangular layout in Figma to ensure the object appeared at the intended position once projected. The technical development of the Skyboxes required extensive iteration. Initial tests revealed significant distortion when flat 2D compositions were wrapped onto a spherical Skybox surface in Unity. To resolve this, projection centers and object placements were recalibrated, and illustrations were repositioned using adjusted polar coordinates. Early tests also revealed color discrepancies between PNG exports and their appearance in VR due to scene lighting. Converting Figma assets from PNG to OpenEXR (openexr.com/en/latest) significantly improved visual accuracy under high dynamic range (HDR, www.adobe.com/creativecloud/photography/discover/hdr.html), which ensured more reliable tone mapping. A light and a dark version of all the Icons were also created to test what works best for our purpose. The final set consists of eleven Skyboxes exported as OpenEXR textures for optimal HDR compatibility. These were integrated into Unity as non-interactive environmental layers with optimized rendering settings to improve performance across standalone Meta Quest headsets.

#### Extraction Site Component

To indicate the spatial origin of a dataset in the human body, each scene features an Extraction Site Component (see Extended Data Fig. 3.c with the extraction site for hubmap-small_intestine-miao-100_microns-10_4 in the duodenum of the small intestine), which shows where a dataset is located with an HRA 3D reference organ, enabled by prior registration of the spatial origin via the RUI. This component imitates the look of the RUI and Exploration User Interface (EUI)^4^ but introduces a district visual style to fit the visual style of the HRA Organ Gallery. While organ models in the HRA typically have materials that aim to imitate their physical look, 3D reference organs in Extraction Site Components are assigned a material that uses a Fresnel shader with transparency and a dark blue albedo color. An overlaid extraction site marker (cuboid) with a red material is always rendered on top, even if located deep within an organ, to indicate the position, rotation, and scale of the extraction site of the dataset. New Extraction Site Components can be created via a Unity Editor script (learn.unity.com/tutorial/editor-scripting), which needs pointers to a 3D reference organ, a cached HRA API response as a *ScriptableObject* (docs.unity3d.com/2022.3/Documentation/ScriptReference/ScriptableObject.html), and a tissue block/sample ID. Details on these are provided in the Native Unity packages and concepts section.

#### Acknowledgments

In each scene, 2D text panels acknowledge the Data Experts and all relevant metadata plus a verbose description of the data presented.

### Shared scripts and Unity features between scenes

#### Native Unity packages and concepts

The HRA Organ Gallery is developed in Unity, which allows developers to implement application data structures and behaviors as custom **components** using **C# classes** (learn.microsoft.com/en-us/dotnet/csharp). A Unity component is an instance of a C# class that is derived from the *Component* base class (typically *MonoBehaviour*, see below) and is attached to a *GameObject* (see below). In this context, the class serves as the blueprint, while the component is the instantiated object that provides functionality to the *GameObject*. During compilation, Unity compiles these C# classes into .NET assemblies (dotnet.microsoft.com/en-us/), which are executed by its.NET-compatible runtime and provide access to the engine’s APIs for application development and visualization. As a production-strength game engine, Unity provides a wide variety of native tools for building real-time 3D applications in VR. Below are the major ones used for the HRA Organ Gallery.

##### Canvas:

This element enables the creation of 2D UIs and UI elements, such as buttons, sliders, and drop-down menus (docs.unity3d.com/2022.3/Documentation/ScriptReference/Canvas.html). They also provide events to detect user input on these elements (such as hovering, clicking, and dragging). Every scene in the HRA Organ Gallery uses at least 1 *Canvas* to display text and other 2D UI elements (such as legends).

##### GameObjects:

These are a central concept and represent entities in a Unity scene. GameObjects can have components such as a *Transform*, which specifies position, rotation, and scale, *MeshRenderer*, which takes a series of vertices and edges (mesh) and enables the user to assign a material to them, and many others, such as physics-based (*RigidBody* and *Collider* for collision detection) or entirely custom ones (all C# classes derived from *MonoBehaviour*). Documentation for the *GameObject* class is available at docs.unity3d.com/2022.3/Documentation/ScriptReference/GameObject.html.

##### MonoBehaviours:

This base class offers lifecycle functions, such as *Awake()* (run before first frame), *Start()* (run on first frame), and *Update()* (run every frame) that allow developers to implement functionality across the runtime of an application. C# fields in a *MonoBehaviour* can be serialized in the Unity Inspector for live-editing during development. Documentation for *MonoBehaviours* is available at docs.unity3d.com/2022.3/Documentation/ScriptReference/MonoBehaviour.html.

##### ScriptableObjects:

These are serialized data containers that allow developers to pass data around the application during development and at runtime. ScriptableObjects are written as C# classes that inherit from the ScriptableObject base class (docs.unity3d.com/2022.3/Documentation/Manual/class-ScriptableObject.html). Instances are created and saved as assets in the project structure of the Unity codebase. *MonoBehaviours* and other C# classes can point to ScriptableObject instances. Here are frequently used ScriptableObjects in the HRA Organ Gallery:

- *SOCellColorMapping*: Contains mappings between cell type labels (strings) and colors (using Unity’s Color struct, see docs.unity3d.com/ScriptReference/Color.html).
- *SOCellPositionList*: For visualizations of cells in 3D space, CSVs with x, y, z-position and cell type labels are ingested as ScriptableObjects (e.g., for sennet-lymph_node-enninful_farzad-1_centimeter-10_2, hubmap-small_intestine-miao-100_microns-10_4, and hubmap-large_intestine-zhu-100_microns-10_4).
- *SODatasetCellTypeFrequency*: Is created together with instances of *SOCellPositionList*; this ScriptableObject type lists cell type labels and their respective number of occurrences within an ingested CSV.
- *SOLevelIndex*: Describes a level with metadata such as scene name, scale, and pointers to assets that need to be loaded, such as Skyboxes and Icons.
- *SOLevelList*: Points to a list of *SOLevelIndex* instances*.* The buttons on the Elevator Panel utilize the *SOLevelList* to determine which level needs to be loaded with Unity’s *SceneManager* library (docs.unity3d.com/2022.3/Documentation/ScriptReference/SceneManagement.SceneManager.html) based on user input.
- *SONodeArrayFromAPI*: Captures HRA API responses with metadata for 3D reference organs and tissue blocks. This is used for hra-whole_body-hra-1_meter-10_0.

##### Socket interactors:

This component can be added to any *GameObject* in the scene and causes another *GameObject* with a *XRBaseInteractable* component to snap in place inside the socket interactor. This can be used to rapidly reset the position, rotation, and scale after the user has interacted with a 3D object (docs.unity3d.com/Packages/com.unity.xr.interaction.toolkit@2.6/manual/xr-socket-interactor.html). Socket interactors are provided by the XR Interaction Toolkit (see below).

##### XR Interaction Toolkit:

This is a collection of components and prefabs (templates) to author user interactions with 2D, 3D, and UI elements in VR, augmented reality (AR), and mixed reality (MR) environments. It provides high-level abstractions for actions, such as hovering, selecting, and activating for a variety of input devices, including VR controllers that the HRA Organ Gallery utilizes. Additionally, it contains the *XRBaseInteractable* and *XRBaseInteractor* base classes, which allow the developers of the HRA Organ Gallery to make organs, datasets, buttons, and other assets interactable. Documentation for XR Interaction Toolkit 2.6, which was used for the HRA Organ Gallery, is available at docs.unity3d.com/Packages/com.unity.xr.interaction.toolkit@2.6/manual/index.html.

##### XRBaseInteractable:

This base class for components allows developers to make a *GameObject* intractable for a user via, e.g, grabbing and rotating it. It defines a series of base events that are raised when the user hovers, selects or activates the *GameObject*, and handles physics-based interactions. Important derived classes frequently used in the HRA Organ Gallery are *XRGrabInteractable* in support of grabbing and moving GameObjects (docs.unity3d.com/Packages/com.unity.xr.interaction.toolkit@2.6/manual/xr-grab-interactable.html) and *XRGeneralGrabTransformer* in support of scaling GameObjects (docs.unity3d.com/Packages/com.unity.xr.interaction.toolkit@2.6/api/UnityEngine.XR.Interaction.Toolkit.Transformers.XRGeneralGrabTransformer.html).

##### XRBaseInteractor:

This base class for components allows developers to enable the user’s VR controller (or other input device) to point at GameObjects and 2D/3D UI elements, e.g., with the *XRRayInteractor* component (docs.unity3d.com/Packages/com.unity.xr.interaction.toolkit@2.6/manual/xr-ray-interactor.html).

#### Custom C# components and prefabs

The HRA Organ Gallery, the following custom C# components were developed and reused across multiple scenes:

##### CellData:

This class is derived from Unity’s *MonoBehaviour* base class. It defines a component that can be attached to any *GameObject* representing a cell while capturing metadata about this cell, i.e. its label and color. This makes this metadata available to the application for querying at runtime.

##### CellLegend:

Also derived from the *MonoBehaviour* base class, this component needs references to a VisualizerBase (or a derived class, see below), a *SODatasetCellTypeFrequency ScriptableObject* (see above), and a *SOCellColorMapping ScriptableObject* to build a legend in 3D space.

##### Dot prefab:

Unity allows the re-use of frequently used 3D objects as so-called prefabs (templates). If the prefab is changed, all instances of the prefab inherit the change. In the HRA Organ Gallery, cells are visualized as 2D circles with a color and *CellData* component that can be assigned or changed at runtime. The *dot prefab* is used in every scene where a VisualizerBase-derived component is used.

##### LegendDisplay:

This class defines a component that allows the developer to point to a vertical layout of legend entries (2D UI elements), a CellLegend (see above), a *SODatasetCellTypeFrequency ScriptableObject*, and a *SOCellColorMapping ScriptableObject*. It then lays out the CellLegend in space with the values from the *SODatasetCellTypeFrequency ScriptableObject* and the provided color mapping from the *SOCellColorMapping ScriptableObject* .

##### OnHoverSendMessage:

This custom class extends *MonoBehaviour* and implements *IPointerEnterHandler* and *IPointerExitHandler*, 2 interfaces from Unity’s EventSystems name space. It is attached to all CellLegend entries. When the user hovers over a legend entry (e.g., a cell type), this component broadcasts the label of the hovered cell type so all cells in the scene are highlighted. This enables brush-and-link functionality using VR controllers.

##### VisualizerBase:

This abstract base class, through its derived classes, allows the developer to point to an empty GameObject (container) in 3D space, define a maximum width in 3D space, a scaling factor, and a color scheme. It also defines abstract functions for preparing the scaling and building a visualization; these functions must be defined in all derived classes, which may implement visualizations differently. 2 derived classes are used in the HRA Organ Gallery: *Visualizer3D* (used in sennet-lymph_node-enninful_farzad-1_centimeter-10_2, hubmap-small_intestine-miao-100_microns-10_4, hubmap-large_intestine-zhu-100_microns-10_4, and hra-multiscale-comparison-codex-100_microns-10_4), which visualizes a list of cell types in 2D or 3D space, and *VisualizerBiomarkers* (used in sennet-brain-phatnani-1_millimeter-10_3), which lays out Visium cell spots on a floor-aligned 2D plane in 3D space and adds vertical line meshes to represent 3D spikes.

#### Editor scripts and *EditorWindows*

Unity ships with an Editor where developers can build complex 3D scenes with hierarchies, inspect project files, run performance analysis with built-in UIs, and test the application using emulation for VR devices. It also allows developers to expand its Editor with Editor scripts (learn.unity.com/tutorial/editor-scripting), which use the UnityEditor namespace (docs.unity3d.com/2022.3/Documentation/ScriptReference/UnityEditor.html) to build custom menus, windows, and inspectors. Developers of the HRA Organ Gallery utilize 2 major EditorWindows:

##### IngestCellPositions:

While Unity applications can natively read CSV through native .NET or third-party CSV libraries at runtime, larger CSV files are best converted to ScriptableObjects to improve speed. The *IngestCellPositions EditorWindow* allows developers to point to a CSV file and ingest it by instantiating 2 ScriptableObjects: a *SOCellPositionList* and a *SODatasetCellTypeFrequency* (see ScriptableObjects list under Native Unity packages and concepts section for both).

##### IngestScene:

This *EditorWindow* enables developers to cache responses from 2 endpoints in the HRA API: apps.humanatlas.io/api--staging/v1/scene (which serves all 81 3D reference organs as of HRA v2.5 and tissue blocks with metadata) and apps.humanatlas.io/api--staging/v1/reference-organ-scene (which serves a user-specified 3D reference organ and its tissue blocks with 3D position, rotation, and scale relative to the origin of the organ as of HRA v2.5). These are saved as ScriptableObjects of type *SONodeArrayFromAPI* (see ScriptableObjects list under Native Unity packages and concepts section), which are then referred to by various scene setup components in hra-whole_body-hra-1_meter-10_0. *IngestScene* is typically run in preparation of every HRA release (June and December every year as of July 2026) to get the latest 3D reference organs and tissue blocks from the HRA and make them available to the application without the need for an internet connection.

#### Elevator Transition Scenes

The HRA Organ Gallery’s Multiscale Elevator System (see Extended Data Fig. 1) includes a cylindrical elevator that allows users to travel between different biological scales, ranging from 10^0^ (whole body level) to 10^−9^ (molecular level). Each Elevator Transition Scene lasts 30 seconds and contains either a human body model for reference (for scales 10^0^ through 10^−6^) or a group of red blood cells in a blood vessel (for 10^−7^ and below). During the scene, the user is placed inside the elevator and moves either down or up, depending on whether they are traveling to a smaller or larger scale. After 30 seconds, the scene routes the user to the selected destination scene. Elevator Transition Scenes can be skipped via a toggle on the Elevator Panel (see red button in the top row of the Elevator Panel in Extended Data Fig. 3.d). A demonstration video is available at cns-iu.github.io/hra-organ-gallery-supporting-information/#videos.

Two major challenges had to be solved to make Elevator Transition Scenes work: relative size and relative speed.

##### Relative size:

In each Elevator Transition Scene, the sizes of both the user in the elevator and the reference object (human or red blood cells) are adjusted such that their scale difference corresponds to the level to which the user travels. For example, if the user travels to a scene on the 10^−4^ level, the human body will be 4 orders of magnitude larger than it would typically appear. Starting at the 10^−7^ level, however, the scale difference (which roughly corresponds to the distance between Chicago, IL, and Cairo, Egypt) becomes so large that the human body becomes unrecognizable and invisible to the user due to the large draw distance. As a result, for the 10^−7^ level and below, a different reference object is used (red blood cells). Extended Data Fig. 1 shows these 2 reference objects from different angles (**a-b** for the human, **c-d** for the red blood cells). Moreover, Extended Data Fig. 1.b and d feature the user’s view from inside the elevator while looking at the adult human and a group of red blood cells, respectively.

##### Relative speed:

A related issue arises when the scale difference between user and reference object becomes increasingly large. To give the user the impression of moving, the relative position between the elevator and the reference object needs to change perceptably. However, at vast scale differences, even fast movement (greater than 100 meters per second) is not sufficient to induce this feeling of motion. Additionally, fast movement can introduce rendering challenges for Unity. To address this issue, a particle system was implemented, where particles emerge from the bottom of the elevator in a circular pattern and move upward with visible trails, which creates the illusion of the elevator moving through the scene. This is also explained in the demonstration video at cns-iu.github.io/hra-organ-gallery-supporting-information/#videos.

### Preprocessing and scene setup

#### hra-whole_body-hra-1_meter-10_0

**Extended Data Fig. 5.**
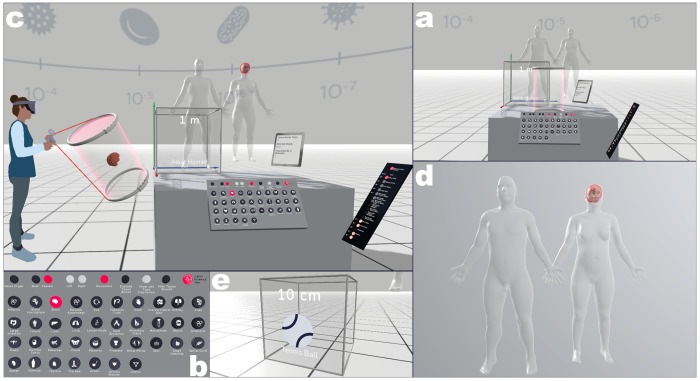
**a,** Overview of hra-whole_body-hra-1_meter-10_0 with work area and Skybox overhead. **b,** Organ selection keyboard with organ and sex selection buttons (brain, female) selected. **c,** The user holding the grab cylinder with the female brain. **d, The** currently selected organ is highlighted inside the male and female reference bodies. **e,** Detail shot of the small scale cube on the left side of the user.

In this default start scene, the user is presented with a work area that includes a desk (see Extended Data Fig. 5.a). In the scene, they can move, rotate, and scale all 81 3D reference organs of the HRA v2.5 (humanatlas.io/3d-reference-library?version=11&organ=All%20organs) as well as tissue blocks registered into them. When loaded, the user is presented with an organ selection keyboard (see Extended Data Fig. 5.b), where they can pick an organ to inspect. Organ selection buttons consist of a base button model with a symbol on top of it using HRA organ icons, see HRA Style Guide^37^ and HRA Design System (docs.humanatlas.io/dev/design-system), and the name of the organ below it. The top row features functionality buttons to reset the organ position, rotation, and scale, a sex selection button (if applicable), a laterality selection button (if applicable), and a button to hide tissue blocks. The latter allows the user to inspect organs whose details are obstructed by overlapping tissue blocks. Buttons to switch between user movement and the user’s ability to “explode” tissue blocks as well as to show cell type populations are present but inactive and will be made active in future releases.

Each button has 3 possible states: ready (button can be pressed), pressed (button is pressed), and locked (button cannot be pressed). Colors from the HRA Design System are assigned based on state. Button states are handled by a C# script that captures user input with events-based C# architecture (learn.microsoft.com/en-us/dotnet/csharp/programming-guide/events) and checks which organ, sex, and laterality combinations are possible. When an organ button is clicked, the organ is loaded into a grab cylinder (e.g., the female brain in Extended Data Fig. 5.c) so it can be moved, rotated, and scaled for anatomical exploration (see the scaled up placenta and umbilical cord in Extended Data Fig. 2.a). The loaded organ has all its tissue blocks, which are retrieved via the HRA API endpoints at apps.humanatlas.io/api#get-/v1/reference-organ-scene and apps.humanatlas.io/api#get-/v1/scene. These API calls are made during development using Editor scripts, and the response is cached as a ScriptableObject in Unity, i.e., a data container with a matching C# class that extends the ScriptableObject class in the Unity code base (see Native Unity packages and concepts section). This allows the application to be updated using the most recent HRA data but without the need for an internet connection at runtime. The code at github.com/cns-iu/hra-organ-gallery-in-vr-preprocessing/tree/main/preprocessing-glb-download is used to download all available 3D reference organs of the most recent HRA release at the time of running the code. Once an organ is loaded in the grab cylinder, it is highlighted inside the whole body of either the male or female body on the table (see Extended Data Fig. 5.d). In addition to the Scale Cube in the back of the table, a smaller scale cube (one order of magnitude smaller) is shown next to the user (see Extended Data Fig. 5.e). It scales up and down if the user scales the selected organ up and down.

The position, rotation, and scale transformation for this scene are prepared with the IngestScene Editor script calling the HRA API (apps.humanatlas.io/api), see Editor scripts and EditorWindows section.

#### sennet-lymph_node-enninful_farzad-1_centimeter-10_2

Like other scenes in HRA: Powers of Ten, the CODEX data for the lymph node in this scene is visualized with the *Visualizer3D* component, which is derived from a VisualizerBase component that defines base functionality to take a list of cell types in 2D or 3D, lays it out in space, and applies formatting as needed with brush-and-link functionality (see **Supplementary Fig. 1** and implementation details in Custom C# components and prefabs section).

#### sennet-brain-phatnani-1_millimeter-10_3

**Extended Data Fig. 6.**
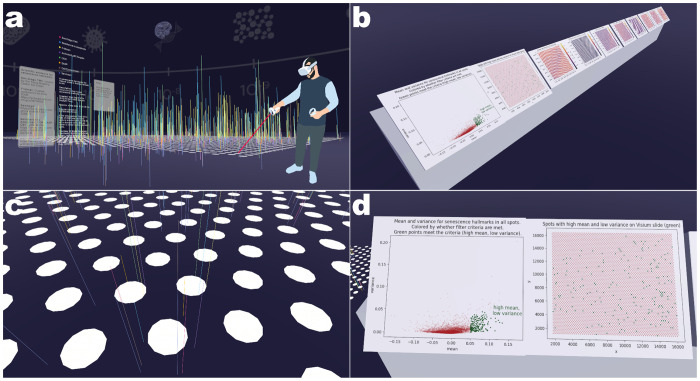
**a,** 3D spike plot for 8 senescence hallmarks (color-coded) above or below the plane of the cell spots in sennet-brain-phatnani-1_millimeter-10_3. **b,** 2D plot gallery with 10 plots. **c,** Detail view of several cell spots, some of them with senescence hallmark expression lines below the plane of the spot. **d,** Detail view of 2 auxiliary graphs (left: mean [x] and variance [y] for senescence hallmark expressions in each cell spot; cell spots in green, i.e., high mean and low variance, get lines for their associated senescence hallmarks; right: the same data but with each cell spots x and y-position in scatter graph).

This scene enables visual exploration of 8 senescence hallmarks across 4,992 spots in Visium data from the middle frontal gyrus of the brain of a 74-year old female, human donor. Relevant SenNet IDs are: SNT239.DNSH.749 (donor), SNT326.PVLT.534 (tissue block), SNT873.QHZM.365 (dataset), and SNT956.RQLW.577 (upload). The scene features 2 major items: a 3D spike plot to visualize expression values for 8 color-coded senescence hallmarks (see Extended Data Fig. 6.a) and a gallery of 10 2D plots made with *plotly* (plot.ly, see Extended Data Fig. 6.b) that were imported as PNG textures, then applied to 2D surfaces in the scene. The user can grab, rotate, and bring these 2D plots with them when stepping into the 3D spike plot.

##### 3D spike plot:

Each dot contains average expressions for 8 senescence hallmarks for all the cells captured in that circular area. If a 3D spike goes above the plane of the cell spot, the corresponding senescence hallmark is positively expressed and below the plane of the spot if it is negatively expressed (see Extended Data Fig. 6.a and c). The 3D spike plot was created using standard UI components in Unity, i.e., dot sprites for 4,992 cell spots on the ground (docs.unity3d.com/2022.3/Documentation/ScriptReference/Sprite.html) and line meshes for the 3D spikes (docs.unity3d.com/2022.3/Documentation/ScriptReference/Graphics.DrawMeshNow.html). To avoid visual clutter in the 3D spike plot, lines are only shown for cell spots that have a low mean and a high variance among their senescence hallmark expressions. As a result, only 1,576 lines are drawn for 197 cell spots; if all were shown, the user would have to navigate through 39,936 lines for 4,992 cell spots.

##### 2D plots:

While the 3D spike plot enables visual and spatial exploration of 8 senescence hallmarks in 3D space concurrently, the gallery of 2D plots presents scatter graphs where color intensity encodes the expression value for exactly 1 hallmark in each plot. 2 additional scatter graphs show which cell spots get senescence biomarkers and where those cell spots are in the Visium grid (see Extended Data Fig. 6.d).

#### hubmap-large_intestine-wong-100_microns-10_4

**Extended Data Fig. 7.**
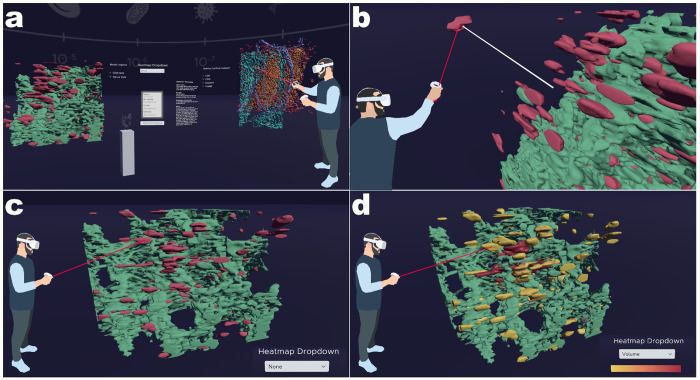
**a,** Overview of light-sheet and confocal microscopy datasets in shared 3D space in hubmap-large_intestine-wong-100_microns-10_4. **b,** The user grabs a cell; a visual tether line (white) is visible. **c,** detail shot of the light-sheet model with dropdown = None. **d,** Same detail shot with dropdown = volume. The color scheme used here is *YlOrRd* from Color Brewer (colorbrewer2.org/#type=sequential&scheme=YlOrRd&n=3).

Two major datasets in v-CyCIF^9^ are explorable in this scene: a light-sheet microscopy dataset and a healthy confocal dataset, both from the large intestine (see Extended Data Fig. 7.a). They are color-coded and share the same color scheme. A legend is shown to the user in the scene. Major pre-processing was done to make the CD8 cells interactable, make visual encoding adjustable by the user, and enable scaling of the light-sheet dataset.

##### Pre-processing

For the light-sheet crop (Extended Data Fig. 7.a**, left**), a quantification table (CSV) and a 3D model with CD8 cells (OBJ) were provided by co-author Wong. While the quantification table had 1 row per cell with an ID, the 3D model listed generic cell names, e.g., "CD8.001", "CD8.002", that did not immediately match the proper CSV identifiers in the quantification table, which also contained the cell positions and volumes. The goal was to determine which 3D object (red cells) corresponded to which cell ID in the data for use in the scene. While manual mapping was initially considered, this proved impractical due to the large number of cells and the fact that many were situated deeply inside the 3D structure, which made them challenging to identify by eye.

###### Matching strategy:

Matching 3D object names to rows in the quantification table involved using both position and volume data. To perform automated matching, the reference systems of the quantification table had to be mapped to the reference system of the 3D model. A Python script was written to automate this mapping process. The key was using 1 easily identifiable cell as a reference point; *Cell_086* (which was manually renamed initially) was chosen as the reference, because it could be easily identified in both the 3D model and CSV data by eye.

###### Unit conversion:

CSV coordinates were in microns, but 3D units were unclear. The script tested multiple conversion factors to handle this unit uncertainty:

- Microns to meters (1e-6)
- Microns to millimeters (1e-3)
- Direct microns (1.0)

###### Coordinate System Offset Calculation:

Using the reference cell, the coordinate system offset Δ_*p*_ was determined as shown in **Equation 1**.

$$\Delta_{p}=p_{3d}-p_{csv}$$

**Equation 1**. The offset was determined by subtracting the x, y, z-coordinates from the quantification table from the x, y, z-coordinates in the 3D model.

###### Automated matching process:

For each cell in the 3D model: (1) The CSV position was converted using the optimal unit conversion and offset. Then, (2) the distance between the 3D cell and its CSV position was calculated. Finally, (3) matches had to pass both position and volume validation.

*bmesh* (a builtin Blender library, see docs.blender.org/api/current/bmesh.html) was used to get precise volumes of the 3D cell meshes while accounting for scaling and transformations. Once a CSV ID was matched to a 3D cell, it was removed from the list of matchable IDs to prevent multiple 3D cells from claiming the same ID.

###### Quality control parameters (Trial & Error):

The tolerance for position errors was set to less than or equal to 3.0 scene units in Blender. The volume error tolerance was set to less than or equal to a 20% difference. Match quality metrics were logged.

###### Algorithm validation:

The script automatically tested different conversion factors and selected the optimal one based on total successful matches. The exemplary output in Table 1 includes detailed match statistics. The resulting mapping was validated with the Data Expert (co-author Wong).

##### Scene setup

Following the cell matching, the 3D model, now with renamed 3D cells, was exported as a FBX file (docs.blender.org/manual/en/2.80/addons/io_scene_fbx.html) and integrated into the Unity scene. Light probes were strategically positioned around the entire 3D model to implement mixed lighting for simulating real-time lighting during runtime. This approach balances visual quality with performance requirements for VR applications. Socket interactors (see Native Unity packages and concepts section) were added for all 3D cells to enable automated snapping functionality: When cells are released within 0.3 meters from their original positions, they automatically return to their designated locations.

In addition to object-level interaction, the scene supports fully free-form VR navigation. Users are not limited to ground-based or planar movement. Instead, they can move forward and backward, side to side, up and down, and freely fly in any direction. This locomotion system allows users to (1) orbit the entire 3D cell structure from the outside, (2) fly through dense regions of the model, and (3) navigate inside the 3D tissue volume itself to inspect internal spatial relationships between cells. One such detail is shown in Extended Data Fig. 2.b.

###### Cell ID-specific socket interactors:

To ensure biological correctness and prevent user error, each cell socket was designed to only accept its corresponding cell. Every cell and socket were assigned a unique cell ID component, derived from the matched CSV identifier. A custom socket interactor enforces strict ID matching, i.e., a socket only allows hovering and selection if the ID of the grabbed cell exactly matches its own. This guarantees that cells cannot be placed into incorrect locations, even accidentally.

###### Cell removal, return logic, and visual tethering:

Early interaction testing revealed a usability issue in that once a cell was removed from its socket, it became difficult for users to remember where that cell originally belonged, especially in dense regions of the structure. To solve this, a visual tethering system was introduced (see Extended Data Fig. 7.b). Each cell is dynamically connected to the centroid of its original socket location via a thick white line. When a cell is grabbed, this line appears between the cell and its home position and is updated in real time as the cell is moved. When the cell is returned to its socket, the tether automatically disappears.

##### Other elements in the scene

###### Data-driven heatmap visualization:

To make the dataset analytically useful, a heatmap system was added to the cells. Minimum intensity, volume, distance from origin, and sphericity can be visualized with color intensity from yellow (low) to red (high) directly on the 3D cells via a dropdown selection (see Extended Data Fig. 7.c–d for an example). Each dataset was imported as structured heatmap data based on the quantification table (using a *ScriptableObject* class, see Native Unity packages and concepts section) and mapped to the corresponding cell IDs. When the user selects a metric from the dropdown menu, cells are colored according to their relative values so users can instantly identify minimums, maximums, and spatial trends across the tissue. This enables spatial exploration and comparison without requiring external plots or tables.

###### UI panel for cell-related information:

A contextual UI panel was added to display precise numerical data for individual cells. When a user grabs a cell, the panel updates in real time to show: Cell position (x, y, z), minimum intensity, volume, and sphericity.

###### Adjacent healthy confocal large intestine model, initial integration:

The adjacent healthy large intestine model was provided by co-author Wong in 4 separate OBJ files representing different biological components: CD8, CD31, Claudin and TUBB3. These 4 files together form a single combined structure with significant spatial overlap and interconnection between layers.

##### Mesh optimization and performance considerations

When initially loaded, the combined light-sheet crop and the healthy confocal large intestine model contained approximately 6 million polygons, which was well above the recommended target of ~1.3–1.8 million polygons for VR scenes (see Performance metrics for Meta Quest VR headsets section). To reduce geometric complexity, all 4 OBJ files for the confocal dataset were imported into Blender and decimated individually with the *decimate* modifier (docs.blender.org/manual/en/latest/modeling/modifiers/generate/decimate.html). A decimation factor of 0.05 was selected through trial and error, which resulted in each mesh retaining approximately 5% of its original polygon count while maintaining acceptable visual fidelity. After decimation, the healthy large intestine model was imported into the existing Unity scene. With both the previously integrated CD8 model and the new large intestine model present, the total polygon count in the scene was reduced to approximately 3 million. Runtime testing showed that the scene continued to run smoothly, no significant frame rate drops were observed, and performance remained acceptable despite exceeding the recommended target polygon budget. During normal usage, not all of the polygons are viewable concurrently.

#### hubmap-small_intestine-miao-100_microns-10_4

**Extended Data Fig. 8.**
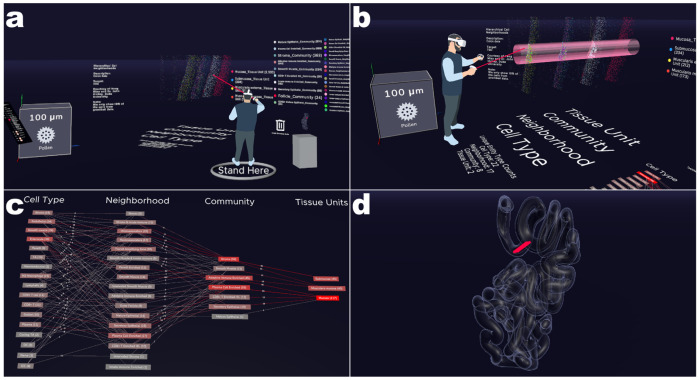
**a,** hubmap-small_intestine-miao-100_microns-10_4 scene with a “Stand Here” sign that directs the user to a good starting point with a view of all features in the scene. **b,** 4 layers of aggregation with a selection cylinder and a text field that shows the number of unique cell types, neighborhoods, communities, and tissue units (similar to what is shown in Extended Data Fig. 2.c). **c,** Overhead screenshot of the alluvial diagram with the number of cells per types, neighborhoods, communities, and tissue units. **d,** Extraction Site Component showing the spatial origin of dataset HBM573.SWGH.988.

Because this scene contains various features in 3D space, a “Stand Here” sign on the ground marks a user’s recommended initial position (see Extended Data Fig. 8.a). 4 layers show the same cells in 4 different colors based on 4 different layers of aggregation: cell type, neighborhood , community, and tissue unit. With their right hand, the user can then place an adjustable, cross-layer selection cylinder to highlight the same cell on all 4 layers, with context provided by a text canvas that shows the number of selected cells per layer (see Extended Data Fig. 8.b and Extended Data Fig. 2.c). A trash can icon allows the user to delete all selection cylinders. Additionally, an alluvial diagram on the ground shows how many cells on which cell type, neighborhood, community, and tissue unit were selected by using color intensity (white to red) and numeric labels (see Extended Data Fig. 8.c). Additionally, an Extraction Site Component highlights the spatial origin of the data in the proximal jejunum of the small intestine (see Extended Data Fig. 8.d). The data^22^ comes from HuBMAP dataset HBM573.SWGH.988 in sample HBM787.FHZM.948 from donor HBM578.DXWB.873.

To implement the text canvas with the number of selected cell instances per layer, a *Canvas* (see Native Unity packages and concepts section) was set up along with several scripts: *CellLogger,* a data processing script, and *CellTypeDisplay*, a script to print the uniquely identified cells per layer of aggregation across as a simple label. *CellLogger* processes the selected data labels into a dictionary grouped by x-y coordinates for each entity, while *CellTypeDisplay* prints the unique entities in the selection cylinder to a label in front of the visualization in the scene.

To create the alluvial diagram, several implementation steps were performed: A *GraphVisualizer* script uses the data grouped by x-y coordinates obtained from the *CellLogger* script to map relationships between the unique entity types into a graph. It also logs the number of instances of each entity in each entity node, and a weight along the graph edges showing the number of entity relationships of that type. The nodes and edges are also color coded to show a gradient of weights as they increase/decrease. The trash can icon was remapped to clear the alluvial diagram on click.

#### hubmap-large_intestine-zhu-100_microns-10_4

This level depicts an early-stage, cancer-afflicted large intestine section reconstructed as a 3D tissue map and overlayed with spatial cell type and RNA transcript data with the goal of enabling users to examine how disease-associated cellular and molecular features are spatially organized within the tissue. Traditional histological analysis relies largely on thin tissue sections, typically approximately 5 microns thick, in which few, if any, cells are captured in their entirety. As a result, 2D imaging often fails to reveal critical aspects of tissue architecture and cellular ultrastructure that become apparent only through 3D analysis. Using Space-map^24^, these limitations were addressed by constructing a 3D tissue model and developing visualization approaches that enable exploration across multiple biological scales, from whole tissues and individual cells to molecular features such as RNA localization.

The hubmap-large_intestine-zhu-100_microns-10_4 scene focuses on stem cells and their differentiated progeny within the colonic crypt-villus axis, which are essential for maintaining normal intestinal mucosal function, as well as on diverse immune cell populations residing in secondary lymphoid structures such as Peyer’s patches. 3D visualization and analysis of these tissue-resident immune cells enable detailed characterization of their spatial organization and interactions within their native microenvironments.

Co-author Zhu provided the 3D tissue reconstruction model and its cell layers plus RNA molecules. The primary objective of this scene was to integrate the spatial data visualization layers derived from co-author Zhu’s dataset directly within the corresponding 3D model. To facilitate exploration of the embedded data, the opacity of the 3D model was reduced to an alpha value of 0.2 to allow users to navigate through both the model and the visualization layers simultaneously. This design emphasizes immersive interaction by displaying only data points within a defined proximity to the user, regardless of their original layer assignment, as the user moves through the environment.

An Extraction Site Component in the scene marks the sigmoid colon as the anatomical structure where the tissue was extracted. The data comes from HuBMAP sample HBM896.ZPRW.239, which was retrieved from HuBMAP donor HBM694.QDFG.746.

**Extended Data Fig. 9.**
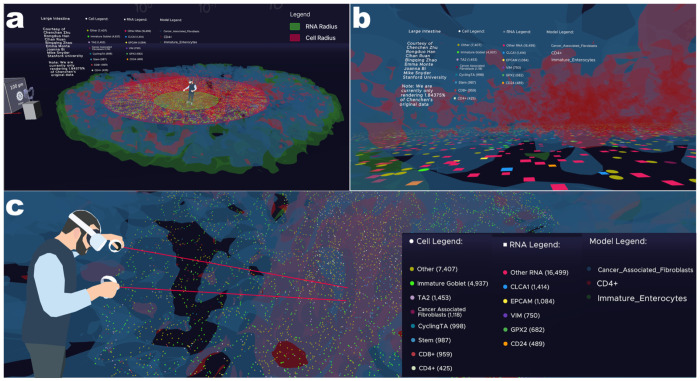
**a,** A user in the combined data of hubmap-large_intestine-zhu-100_microns-10_4. Note the differing radii for cells and RNA in support of performance in VR as well as the similarity to the typical rendering of a disk-shaped galaxy, such as the Milky Way. **b,** A low-angle view of cells and RNA within the reconstructed 3D model. **c,** A user pointing towards cells and RNA molecules with the ray interactor on their VR controllers.

An overview of this scene is presented in Extended Data Fig. 9. This scene posed 2 major technical challenges:

##### Data integration:

It contains a 3D model (OBJ), 29 layers of cells in 2D (CSV), and, for each of these layers, RNA molecules (CSV). These 3 data types had to be aligned in the same virtual space. The guiding principle for the visual style of this scene was the typical rendering of a disk-shaped galaxy, such as the Milky Way (see Extended Data Fig. 9.a).

##### Performance:

The original 3D model already had 1.6 million polygons. Each of the 29 cell layers contained around 80,000+ cells, which, if all 29 layers were rendered, would amount to 2.32 million cells in a singular scene, which would be too computationally expensive on the Meta Quest hardware. The scene exemplarily shows cell layer 16 (18,285 cells) with RNA layer 16 (20,919 RNA molecules) as these are roughly in the center of the z-axis (depth) of the 3D model. To address this challenge, the *decimate* modifier in Blender was used to reduce the polygon count by ~41% to 942,436. Additionally, a visibility radius around the user ensures that only cells and RNA molecules within 300 (cells) and 100 microns (RNA) are rendered, respectively, see Extended Data Fig. 9.a. A detailed view of cells and surrounding RNA molecules is available in Extended Data Fig. 9.b. An illustration of a user traversing the data alongside the legend is shown in Extended Data Fig. 9.c. Extended Data Fig. 2.f shows an alternative angle on a user exploring a local cluster of cells and RNA molecules.

##### Data preprocessing:

Generating visualization layers required extensive preprocessing. While originally all 29 individual CSV files (with native 2D coordinates) were downloaded and given a z-position with an iterative z-axis offset of 0.03 meters in VR to fill out the depth of the 3D model, the deployed hubmap-large_intestine-zhu-100_microns-10_4 focuses on cell layer 16 and RNA layer 16 based on co-author Zhu’s input. On these layers, cell and RNA molecule labels were aggregated to the top-5 plus an “other” category to reduce the number of colors used in the visualization (cells: Immature Goblet, TA2, Cancer Associated Fibroblasts, CyclingTA, Stem; RNA: CLCA1, EPCAM, VIM, GPX2, CD24). These 2 CSV files were then processed using the *IngestCellPositions* Editor script in Unity. This made the combined CSV file more easily usable at runtime and editable in the Unity Inspector. For this combined CSV file, the ingest process generated 2 *ScriptableObjects*: The first *ScriptableObject* stores the spatial coordinates (x, y, z-positions) of all cells across all layers with their corresponding cell type label. The second *ScriptableObject* contains summary statistics describing the frequency of each unique cell type per layer. These data structures serve as the primary inputs for subsequent visualization.

##### Visualization:

3D visualizations are generated using the *Visualizer3D* component, which uses both these *ScriptableObjects* produced during data preprocessing (see Custom C# components and prefabs and Native Unity packages and concepts sections). While rendering, the application instantiates individual GameObjects representing cells with *CellData* components and places them at spatial locations defined by the coordinate data stored within each layer’s *ScriptableObject*. Cell labels and frequencies are obtained from the corresponding *_frequency ScriptableObject*. The resulting visualizations are then positioned within the Unity scene using appropriate z-axis spacing to ensure accurate alignment with the reconstructed tissue model. As a result, the final scene combines the 3D tissue reconstruction model with its corresponding spatial cellular data. To accommodate the sheer volume of cells, the *IngestCellPositions Editor* script was set to ingest only 1/64^th^ of the original number of cells, making it comfortable to render 18,285 cells and 20,919 RNA molecules of layer 16 with the targeted hardware.

#### cifar-liver-bader_xing-100_microns-10_4

**Extended Data Fig. 10.**
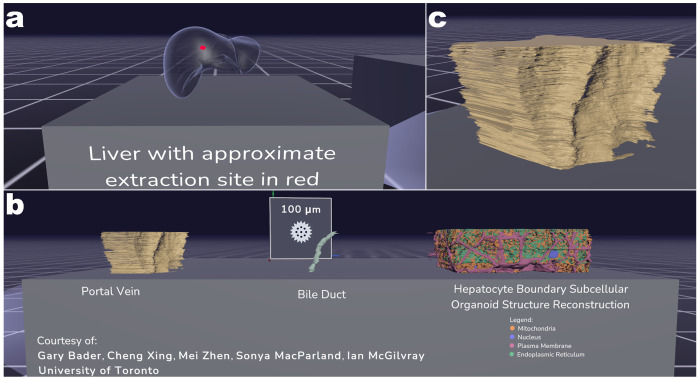
**a,** Extraction Site Component for cifar-liver-bader_xing-100_microns-10_4 (periportal region of the liver). **b,** Portal vein, bile duct, and hepatocyte organelle structures on a table next to each other, each of them grabbable, movable, rotatable, and scalable. **c,** Detail view of the portal vein. Future work aims to improve the sectioning artifacts visible on the surface of the model.

The data for this scene comes from the caudate lobe of the liver’s periportal region (see extraction site in Extended Data Fig. 10.a.). 3 models were shared and pre-processed (Extended Data Fig. 10.b). A detailed view is available for the portal vein (Extended Data Fig. 10.c), bile duct (Extended Data Fig. 2.d), and hepatocyte organelle structures (Extended Data Fig. 2.e).

##### Pre-processing

Co-authors Bader and Xing shared 3 TIFF files containing slice data of cross sections for portal vein, bile duct, and hepatocyte organelle structures. As received, they were not ready for VR, since they lacked mesh-based surfaces to render. The SOP for adding new datasets to the HRA Organ Gallery^12^ outlines explain 2 methods to generate 3D models (FBX files) from these TIFF files:

1. Use the Microscopy Nodes^26^ plug-in (extensions.blender.org/add-ons/microscopynodes) in Blender to load TIFF files, then export them as FBX files.
2. Process TIFF files into STL files using Fiji (imagej.net/software/fiji/), then convert STL to FBX in Blender and reduce polygon count with MeshLab (www.meshlab.net).

While method #1 is the preferred method to convert these TIFFs into FBX for usage in Unity, for this scene, it preserved too much of the surface definition in the FBX, which led to a surplus of polygons for the Meta Quest 3 VR headset (see Performance metrics for Meta Quest VR headsets section). While Blender’s *decimate* modifier can reduce polygons in 3D models, it is not effective for large models with many internal structures. To reduce polygon counts effectively while preserving most of the surface definition, method #2 was used.

##### Scene setup

An Extraction Site Component (see Extended Data Fig. 10.a) shows the spatial origin of the liver sample from which the TIFF files (and FBX files after conversion) were generated. A cuboid table displays the portal vein, bile duct, and hepatocyte organelle structures (see Extended Data Fig. 10.b). To enable interaction, an *XRGrabInteractable* was added to each of them for movement and rotation, along with a *XRGeneralGrabTransformer* to enable scaling (see Native Unity packages and concepts section). For aesthetic reasons, the model materials were adjusted to appropriately different but muted colours that are aligned with the HRA Design System.

#### hra-multiscale-comparison-codex-100_microns-10_4

This scene is set up to convey to users how the same cell type distribution can exist in multiple biological entities (entire lymph node and proximal jejunum) at different scales using the CODEX data from sennet-lymph_node-enninful_farzad-1_centimeter-10_2 and hubmap-small_intestine-miao-100_microns-10_4 (see **Supplementary Fig. 2**). Since the main focus of this scene was to visualize common cell types across 2 scales, a common cell type color map was needed. Related HRA work^27^ on crosswalking and aggregating cell to higher-level cell types using Cell Ontology (CL)^38^ was used; the CSV file with these higher-level cell types is available on GitHub (github.com/cns-iu/hra-cell-distance-analysis/blob/1f73eed7032af63a6174b52bd0a8057e38c051df/data/mapping_files/generated_cell_type_complete_crosswalk.csv).

To facilitate comparison across shared, higher-level cell types, the following steps were needed:

1. The original CSV files with 3D cell positions and types were enriched with additional columns to represent level 3 (original, lowest aggregation), level 2 (medium aggregation), and level 1 (highest aggregation) cell types.
2. A mapping from cell type to color was created to standardize colors across the common cell types. This was done via an Editor script. A *ScriptableObject* was used to make the color map available at runtime and editable in the Unity Editor (see Shared scripts and Unity features between scenes **section** for technical details).
3. Both datasets were visualized in the scene, but the width of the lymph node was increased by 2 orders of magnitude from the user’s perspective, because it is 2 orders of magnitude larger than the slice of proximal jejunum. In their processed form, both datasets share the same color mapping.
4. The HRA 3D reference organ for the male skin (humanatlas.io/3d-reference-library?version=11&organ=Skin) was placed in the scene with the correct scaling difference to the lymph node.
5. Finally, to convey the scale difference between the 2 biological data visualizations and the 3D reference organ with regards to a known real-world object, a 3D model of Burj Khalifa, the tallest building on earth as of July 2026 at a height of 882 meters, was added to the scene in its true (non-scaled) size; a flat plane attached to its summit served as visual reference for its height of 882 meters. This model does not change scale.
6. Next, *XRGrabInteractables* and *XRGeneralGrabTransformers* (see Native Unity packages and concepts section) were added to both visualizations to allow the user to scale the spatially arranged data visualization. A scale synchronizer script on an empty GameObject synchronizes the scale of the proximal jejunum data, the lymph node and the HRA 3D reference organ for the male skin, to show how they scale uniformly together.
7. Finally, a reset button next to the cell type legends enables users to reset the visualizations to their position, rotation, and scale.

#### Performance metrics for Meta Quest VR headsets

As of July 2026, Meta provides performance targets for testing and analysis during development for Meta Quest VR headsets at developers.meta.com/horizon/documentation/unity/unity-perf. This comes in the form of recommended draw calls (i.e., commands sent from the CPU to the graphics API requesting that the GPU draw an object on the screen) given light, medium, or busy simulations (i.e., the number of active graphical elements in the scene). While no concrete metrics are provided for what constitutes light, medium, or busy simulations, it can reasonably be assumed that, despite the high-quality 3D data, the scenes presented in this paper constitute at most medium simulations due to the low number of 3D objects on screen, the simple environment, the lack of background elements and animations, the use of baked or mixed lighting instead of real-time lighting, and the lack of post-processing effects. Meta recommends 400-600 draw calls maximum for these scenes. Likewise, in terms of polygon counts, for the Meta Quest 3 and 3S, 1.3-1.8 million in the user’s field of view are recommended.

## Extended Data

## Figures and Tables

**Fig. 1.**
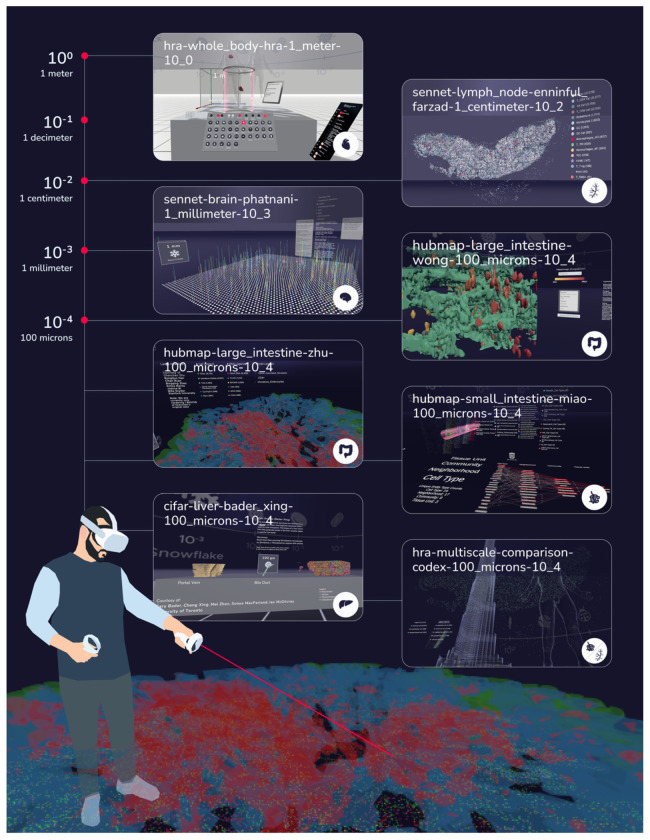
Overview of all scenes in HRA: Powers of Ten. VR enables Data Experts to perform HRA-based data harmonization and visualization across scales. Shown are screenshots from 8 scenes featured in this paper; these screenshots are connected to the corresponding level on the Multiscale Elevator System (on the left). At the bottom, a user is exploring a from the reconstructed 3D tissue map in the large intestine from hubmap-large_intestine-zhu-100_microns-10_4.

**Table 1.** Exemplary mapping results.

3D cell: CD8.001 → Quantification table ID: cell_023.	
Position distance	1.24 units
Volume difference	12.3%
Blender volume	0.001234
CSV volume	0.001398

## Data Availability

A companion website is available at cns-iu.github.io/hra-organ-gallery-supporting-information. To improve documentation and enable readers without VR headsets to learn about the scenes in HRA: Powers of Ten (incl. one documenting Elevator Transition Scenes), videos were compiled at cns-iu.github.io/hra-organ-gallery-supporting-information/#videos, which feature narration by the Data Expert(s) and the Organ Gallery Lead in a recorded VR demo. A workshop to jumpstart pilot project integration was held at the Bioinformatics and Computational Biosciences Branch at National Institute of Allergy and Infectious Diseases and is documented at cns-iu.github.io/workshops/2024-10-24-jumpstart-workshop. High-resolution versions of all figures are available at github.com/cns-iu/hra-organ-gallery-supporting-information/tree/main/hi-res-figs. Supporting information for this paper is available at github.com/cns-iu/hra-organ-gallery-supporting-information. 81 3D reference organs of HRA v2.5 are available at humanatlas.io/3d-reference-library and 3d.nih.gov/collections/hra. Datasets SNT873.QHZM.365 and SNT832.RKFV.643 with their entire provenance are available at data.sennetconsortium.org. Datasets HBM573.SWGH.988 and HBM896.ZPRW.239 with their entire provenance are available at portal.hubmapconsortium.org. The model of Burj Khalifa for hra-multiscale-comparison-codex-100_microns-10_4 was downloaded from sketchfab.com/3d-models/free-burj-khalifa-dubai-c1d6f5884c9c4a56b8d8f9c5555f1902; it was made with Blender 2.90.

## Code availability

The application is available for Meta Quest 2, 3, 3S, and Pro headsets on the Meta Store: https://www.meta.com/en-gb/experiences/hra-organ-gallery/5696814507101529. All code is accessible at github.com/cns-iu/hra-organ-gallery-in-vr. Preprocessing code for this paper is available at github.com/cns-iu/hra-organ-gallery-in-vr-preprocessing.
